# Comprehensive review on the novel immunotherapy target: Leucine-rich repeat-containing 8A/volume-regulated anion channel

**DOI:** 10.7150/ijbs.95933

**Published:** 2024-07-15

**Authors:** Yaohua Hu, Jing Qin, Yifan Ma, Runze Yang, Xinyu Liu, Changhong Shi

**Affiliations:** 1 Division of Cancer Biology, Laboratory Animal Center, The Fourth Military Medical University, Xi'an, Shaanxi 710032, China.; 2 Department of Pathology, Affiliated Hospital of Yan'an University, Yanan, Shaanxi 716000, China.; 3 Gansu University of Traditional Chinese Medicine, Lanzhou 730030, China.; 4 School of Basic Medical Sciences, Medical College of Yan'an University, 580 Bao-Ta Street, Yanan, Shaanxi 716000, China.

**Keywords:** leucine-rich repeat-containing 8A (LRRC8A), volume-regulated anion channel (VRAC), immunotherapy, tumor, virus

## Abstract

Leucine-rich repeat-containing 8A (LRRC8A) is a key component of the volume-regulated anion channel (VRAC) that influences essential homeostatic processes in various immune cells. These processes include the regulation of cell volume and membrane potential and the facilitation of the transport of organic agents used as anticancer drugs and immune-stimulating factors. Therefore, understanding the structure-function relationship of LRRC8A, exploring its physiological role in immunity, assessing its efficacy in treating diseases, and advancing the development of compounds that regulate its activity are important research frontiers. This review emphasized the emerging field of LRRC8A, outlined its structure and function, and summarized its role in immune cell development and immune cell-mediated antiviral and antitumor effects. Additionally, it explored the potential of LRRC8A as an immunotherapeutic target, offering insights into resolving persistent challenges and future research directions.

## 1. Introduction

Ion channels, serving as vital cellular gateways, have consistently been a focal topic in life sciences and drug research and development. They affect numerous crucial biological processes and are associated with various human diseases 1-3. Volume-regulated anion channels (VRAC) are ubiquitously expressed in vertebrate cells and are involved in multiple physiological and cellular processes, including fluid secretion, glutamate release, membrane potential regulation, cell proliferation, migration, and apoptosis 4-6. Despite a thorough understanding of VRAC's biophysical properties, its molecular identity has remained elusive for approximately three decades. Recent breakthroughs, by Voss *et al.* and Qui *et al.*, revealed that the leucine-rich repeat-containing 8A (LRRC8A) protein encoded by the *LRRC8A* gene is a key component of VRAC 7,8. Furthermore, various tissue abnormalities and lethality observed in *LRRC8A*-deficient mice highlight the significance of *LRRC8A* 9,10. Since its first mention, *LRRC8A* has been the subject of numerous studies investigating its electrophysiological characteristics 4,5, pharmacology 5,11, activation and regulatory mechanisms 5,12,13, cell biological functions 4,6, and potential physiological and pathological 4-6,14,15 effects across diverse tissues and cell types. However, research on the relationship between *LRRC8A* and immunity is still in the preliminary exploration stage.

In 2003, Sawada *et al.* discovered an allelic mutation in the *LRRC8* gene in patients with congenital immunoglobulin deficiency, resulting in abnormal development of B cells in the peripheral blood. This was attributed to the translocation of the *LRRC8* gene on chromosome 9, resulting in the loss of 91 amino acids at the C-terminus and abnormal coding of 35 amino acids in the intron region. It is suggested that *LRRC8* may be a potential immune-related gene 9,16. *LRRC8A^-/-^*mice were generated to assess the role of LRRC8A in lymphocyte development and function 10. They exhibited partial hindrance in B cell development, while maintaining intact intrinsic B cell function. Additionally, to eliminate the potential impact of external factors on *LRRC8A*^-/-^ mouse lymphopenia, the authors generated a chimeric *LRRC8A*^-/-^→*Rag2*^-/-^ mouse model by transplanting *LRRC8A*^-/-^ bone marrow cells into *Rag2*^-/-^ mice, that do not produce T and B lymphocytes. Conversely, both *LRRC8A*^-/-^ and *LRRC8A*^-/-^→*Rag2*^-/-^bone marrow chimeras exhibited obvious cell-intrinsic hindrance in early thymic development, with reduced thymocyte proliferation, increased apoptosis, and impaired peripheral T cell function 10. In 2017, a spontaneous hypomorphic mutation in *LRRC8A* known as ébouriffé (ebo/ebo), was reported 17. This mutation resulted from a 2-bp deletion presumed to truncate the 15C-terminal leucine-rich repeats (LRRs) of LRRC8Aebo/ebo mice. These mice exhibit certain similarities to LRRC8A knockout (KO) mice such as curly hair, infertility, reduced longevity, and kidney abnormalities. However, unlike LRRC8A-/- mice, ebo/ebo mice exhibited normal T-cell development and function and an acute antibody response to T-dependent antigens. However, a significant reduction in VRAC current was observed in T cells. Therefore, the activity of VRAC may not be an important factor affecting the development of T cells. In fact, VRAC is composed of five isomers of LRRC8A-E. Knockout of LRRC8A only reduces the activity of VRAC, but it is not completely eliminated. Maybe the reduced or residual VRAC activity also affect the function of T cells, although this effect is not significant 17. Although the underlying mechanisms require further assessment, both human and mouse studies strongly indicate an essential role for LRRC8A in the development and function of lymphocytes.

With advances in research, the critical roles and molecular mechanisms of LRRC8A in immune cells have gradually been elucidated. This review focuses on clarifying the structural and biological characteristics of the LRRC8A/VRAC channel, highlighting recent discoveries associated with its role in regulating T-cell activation and differentiation. Additionally, we explored the role of LRRC8A in inflammatory diseases and its potential oncogenic mechanisms. Furthermore, we assess how the alterations in VRAC activation or inhibition affect the immune response and explore the possible use of VRAC agonists or inhibitors as immunotherapy adjuvants. Finally, we analyzed the mechanism by which LRRC8A regulates immunity to identify novel targets for immunotherapy.

## 2. The structure and biological characteristics of the LRRC8A/VRAC

**Figure 1:**
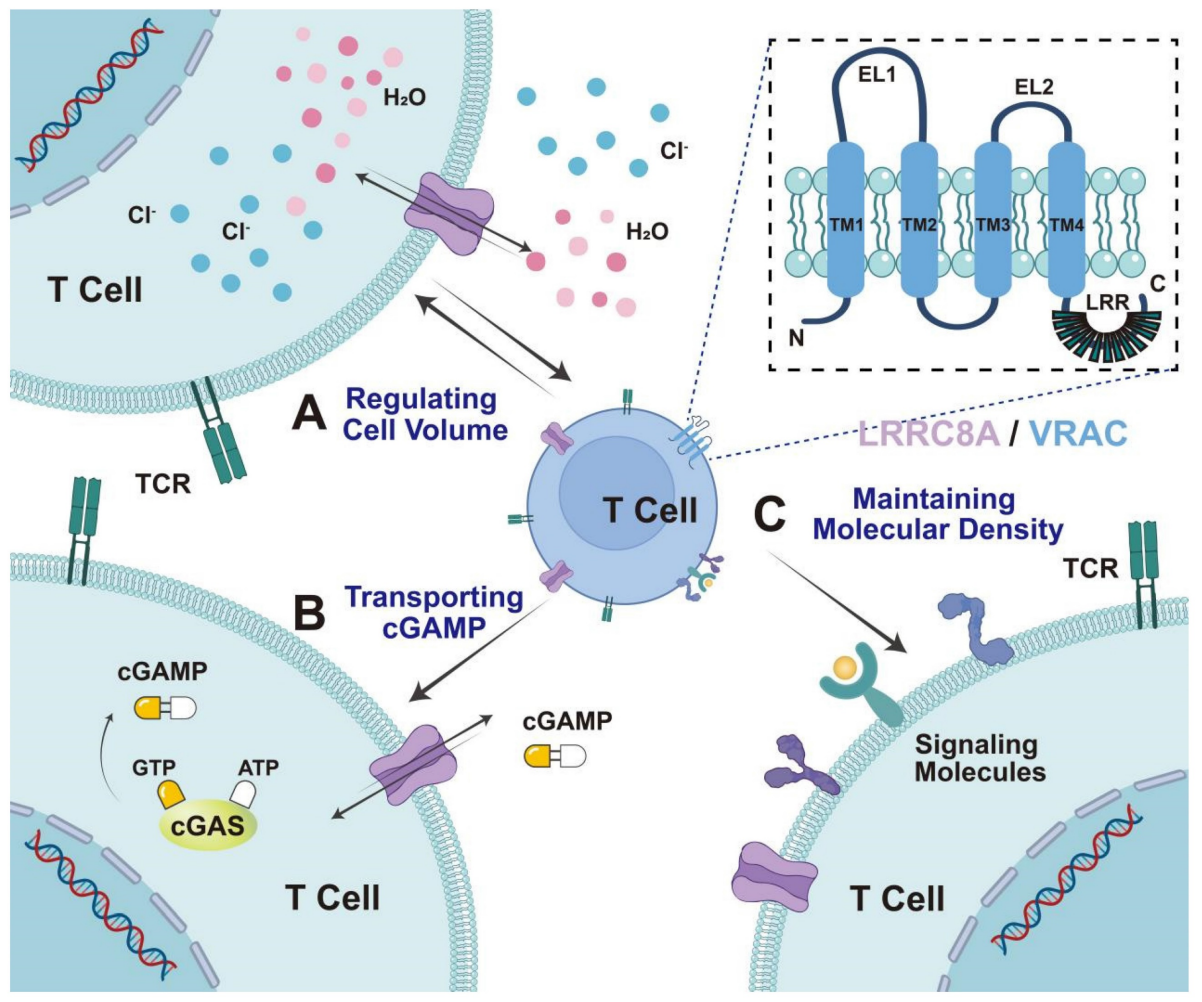
**The structure and biological characteristics of the LRRC8A/VRAC.** LRRC8A comprises four transmembrane domains within the cell membrane. Its biological characteristics in T cells can be divided into three parts: A) regulating cell volume. LRRC8A mediates chloride efflux during cell swelling and regulates cell volume. B. Transporting cGAMP. LRRC8A acts as a transport channel for immune transmitters, including cGAMP, cisplatin, and GTPγS, exerting immune function. C. Maintenance of molecular density. LRRC8A controls TCR signaling by maintaining molecular density at a relatively constant level, preventing an excessive decline in density, and promoting signal transduction.

In the 1990s, several electrophysiological studies reported at least three distinct osmotically activated chloride ion (Cl^-^) channels in human T lymphocytes: γ-aminobutyric acid type A (GABA-A), cystic fibrosis transmembrane gene, and LRRC8 18,19. These channels play a crucial role in T cell development and function. LRRC8, in particular, is well-characterized for its physiological role in volume regulation in hypotonic environments 4. Alterations in the osmotic pressure inside and outside the cell can result in the free movement of extracellular water molecules across the cell membrane or through aquaporin water channels. Conversely, anion channels and other transport proteins on the cell membrane allow intracellular ions and small molecule metabolites to flow out of the cell, thereby regulating volume changes related to cell expansion. Thus, LRRC8 is also referred to as VRAC or swelling-activated Cl^-^ channels 4,20. The fundamental role of the VRAC in regulating cell volume and maintaining a relatively constant cell volume is intrinsically associated with critical cellular processes, including proliferation, development, apoptosis, and cytokine secretion 4,15 (Figure 1).

LRRC8 is formed by assembling LRRC8A and four homologous family members (LRRC8B-E) into a hexamer transmembrane channel protein complex. Furthermore, the subunit composition is cell type-specific and physiologically relevant, as it dramatically affects the activation mechanism, particularly substrate selectivity 21. The LRRC8 family gene spans approximately 2400 bp, contains approximately 810 amino acids, and expresses a protein of approximately 100 kDa. High-resolution cryo-electron microscopy (EM) studies have revealed the structures of LRRC8 subunits, demonstrating that they includes N- and C-termini, four highly conserved transmembrane domains, two extracellular loop domains (EL1, EL2) and a single intracellular loop (IL). Initially, *Leucine-rich repeats (*LRRs) were anticipated to function as ligand-binding domains outside the cells 22,23. However, immunofluorescence staining, performed without damaging the cell membrane, confirmed that the N- and C-termini are oriented towards the inside of the cell 7,8. (Figure 1). This could mean that they have other functions inside the cell. Additionally, the N-terminus of LRRC8A is known to be involved in the formation of the ion conduction pathway of the channel and could affect its selectivity. Mutations at the N-terminus affect VRAC activity and ion selectivity. Moreover, the C-terminus of LRRC8 is essential for channel function, containing multiple conserved sequences and domains related to protein-protein interactions, and playing a crucial role in channel assembly, localization, and regulation. The LRR domain functions as a ligand-binding site, which has implications for channel gating and ion selectivity. The N- and C-termini of the LRRC8 protein play critical roles in channel assembly, function, and regulation, providing important structural and functional information for understanding the physiological and pathological roles of VRAC channels 24-25. LRRC8A and at least one additional LRRC8 paralog are required for VRAC activity 7. Even when other LRRC8 members are overexpressed, LRRC8A KO eliminates chloride currents and swelling of human CD4^+^T cells 8. Therefore, LRRC8A is considered the most crucial component of the VRAC 7,8.

VRAC is widely expressed in mammalian cells and regulates cell volume by mediating intracellular Cl^-^-efflux. Additionally, VRACs are involved in a series of physiological processes such as cell proliferation, differentiation, cell migration, and apoptosis. LRRC8A/VRAC acts as a transport channel for chemotherapeutic agents and immune transmitters. Hypotonic cell swelling, cisplatin, and GTPγS, and the cytokines tumor necrosis factor (TNF) and interleukin-1 (IL-1), have been demonstrated to enhance VRAC activity, facilitate the cellular translocation of extracellular cyclic guanosine monophosphate-adenosine monophosphate (cGAMP) and its analogs (2'3'-cGSASMP and 2'3'-CDAS), enhance stimulator of interferon genes (STING)-dependent inborn errors of interferon (IFN) response, and facilitate immune function 26-29 (Figure 1). The following sections summarize the role of LRRC8A in T cell development, T cell-mediated immune responses, and tumor adjuvant therapy.

## 3. LRRC8A/VRAC regulation of T cell development and immunity

To explore the role of LRRC8A in adaptive immunity, Kumar *et al.* generated mice with systemic LRRC8A KO 10. These mice exhibited higher mortality rates after birth. Further studies revealed that LRRC8A is highly expressed in thymocytes. *LRRC8A* deficiency hinders thymic development, inhibits immature lymphocyte proliferation, accelerates apoptosis, and reduces the number of mature T lymphocytes in the peripheral blood, thereby severely impairing their function. To assess the underlying mechanism, they found that *LRRC8A* KO resulted in the death of thymus cells. Subsequently, they established that the protein kinase B (AKT) signaling pathway plays a crucial role in thymus development. LRRC8A induces AKT phosphorylation and mediates its activation, resulting in T lymphocyte proliferation and development via the lymphocyte-specific protein tyrosine kinase-zeta-associated protein 70- GRB2-associated-binding protein 2-phosphoinositide 3-kinases signaling pathway 10. Additionally, they demonstrated that lack of LRRC8A results in impaired T-cell development associated with impaired thymic development. Therefore, LRRC8A directly affects thymocytes, facilitating their proliferation, and translocating them to the thymus to support their development. Furthermore, LRRC8A participates in T cell metabolism, survival, and memory cell production and enhances the thymic microenvironment to facilitate thymic development.

LRRC8A regulates T-cell volume and affects their function. A 2023 study reported that optimal T-cell activation relies on cell volume regulation 30. Inhibiting VRAC and deleting LRRC8A channel components hindered T-cell activation and function. Moreover, LRRC8A-mediated cell volume regulation is essential for T cell-mediated antiviral immunity. LRRC8A mainly mediates T cell antigen receptor (TCR) signal transduction by regulating cell volume; specifically, the TCR signal initiates substantial anabolic biosynthesis, which increases the intracellular osmolarity and creates osmotic potential, thereby leading to water influx, followed by activating the LRRC8A-formed VRAC to mediate the regulatory volume decrease (RVD). Subsequent RVD is crucial for maintaining TCR signal strength by retaining the molecular density necessary for the signaling machinery. These results indicate that LRRC8A functions as a valve that regulates cell volume and facilitates T-cell activation 30. Furthermore, the activation, migration, and cytokine release of T cells can affect the T cell response, therefore, whether LRRC8A is involved in these processes is worth discussing.

Although *LRRC8A* KO impairs lymphoid development in mice, its direct correlation with LRRC8A/VRAC ion channel activity remains unclear. Platt *et al.* discovered that the VRAC ion access activity in ébouriffé (ebo/ebo) mice was significantly reduced. However, the development and function of T lymphocytes in the (ebo/ebo) mice demonstrated a normal and complete antigen presentation response 17. However, T lymphocyte development and function in these mice were normal, with a complete antigen presentation response 17. This review indicates that LRRC8A results in T lymphocyte developmental defects, independent of LRRC8/VRAC ion channel activity. Although the specific mechanism requires further exploration, data from studies on humans and mice suggests that LRRC8A is crucial for lymphocyte development.

LRRC8A, a VRAC subunit, may also play a role in T-cell expansion in vitro and in regulating T-cell development. Splenic CD3^+^ T-cells from *LRRC8A*^-/-^ mice failed to proliferate in vitro following anti-CD3 or anti-CD3/CD28 stimulation. However, cell proliferation following treatment with phorbol myristate acetate plus ionomycin was normal. The decrease in splenic CD4^+^CD62L^lo^CD44^hi^ T effector memory cells in *LRRC8A*^-/-^ mice indicates that LRRC8A is crucial for peripheral T cell expansion and function 10. Additionally, several studies have confirmed the role of LRRC8A in facilitating T-cell development and proliferation 10,17.

## 4. Application of LRRC8A/VRAC in antiviral immunity

### 4.1 Transporting cGAMP to exert antiviral effects

During innate immune defense, biological resistance to pathogen and microbial invasion and infection requires the cooperation of various immune cells. For example, in the immune response, various immune cells (such as macrophages, T-cells, B-cells, and natural killer cells) communicate by secreting cytokines, expressing surface molecules, and collaborating to resist pathogens 31,32. In virus-infected cells, cGAMP can be transported from infected cells to nearby cells, activating STING and inducing IFN production, thereby serving as an early warning system to antagonize the viruses 33,34. Despite the significant therapeutic potential of cGAMP and other cyclic dinucleotides (CDNs) in antiviral therapy, their negative charge prevents them from effectively penetrating the cell membrane, requiring transport proteins for effective spread 35,36. However, research on the entry of cGAMP into target cells is limited. LRRC8A, a classic channel protein, regulates channel activity and cell volume, directly or indirectly affecting T-cell signaling, and exerts antiviral effects during viral infections. Thus, LRRC8A is expected to emerge as a novel target for antiviral therapy.

In 2020, Zhou *et al.* identified the role of LRRC8/VRAC ion channels in the innate immune response of the host antiviral immunity 26. Moreover, they discovered that deficiency of LRRC8A subunit reduced the potential of host cells to resist herpes simplex virus 1(HSV-1) infection. Further assessment of the cyclic GMP-AMP synthase (cGAS)-STING signaling pathway induced by HSV-1 revealed that the dsDNA- and cGAMP-activated STING signaling pathways in LRRC8A KO cells did not differ significantly from those observed in wild-type cells. However, VRAC activation under low osmotic pressure reduced cGAMP-induced downstream interferon regulatory factor 3 phosphorylation in LRRC8A KO cells. By further assessing the amount of cGAMP entering cells over a short period, researchers discovered that the absence of LRRC8A reduced cGAMP accumulation in cells, inducing a weakened STING signal. Additionally, the infection of exogenously cherry-labeled STING cells was detected by monitoring the expression of GFP-labeled viruses. Through observation of cherry signals, aggregation of STING was detected in virus-infected and the surrounding uninfected cells. However, in LRRC8A-deficient cells, STING aggregation was observed only in virus-infected cells. Furthermore, separating virus-infected and non-virus-infected cells using Transwell assays revealed that STING activation occurred in non-virus-infected cells but was absent in LRRC8A-KO cells. This indicates that LRRC8A/VRAC facilitates the transmission of cGAMP to bystander cells, triggering an augmented IFN response. Thus, LRRC8A is a crucial conduit of cGAMP during cell-to-cell transmission, emphasizing its significance in antiviral immunity 28,37.

Lahey *et al.* also discovered that LRRC8A/C is a direct and bidirectional transporter of cGAMP in human vascular endothelial cells during whole-genome CRISPR KO screening 29. Moreover, the LRRC8D subunit inhibits cGAMP transport. Using pharmacological and electrophysiological methods, researchers discovered that cGAMP flux occurs when the electrochemical gradient changes following LRRC8A channel activation 29. Therefore, LRRC8A mediates the transport of numerous signals and enhances communication between cells, thereby playing an indispensable role in antiviral therapies.

### 4.2 Regulating T cell volume to exert antiviral effects

During T-cell activation, intracellular osmolytes significantly increase owing to ion influxes and biosynthetic processes, including RNA transcription, protein synthesis, and small organic metabolite production 38,39. The accumulation of these osmolytes creates higher osmolarity in the cell, resulting in water influx and enlargement of T-cell volume 40. Swollen cells with VRACs actively regulate cell volume through RVD and modulate the innate immune response. This regulation is also relevant to antiviral therapy.

A previous study generated a T-cell-specific LRRC8A KO mouse model (*LRRC8A*^f/f,Cd4-Cre^) to determine the role of LRRC8A-mediated intrinsic cell volume regulation in T-cell activation and function 30. It was observed that during T-cell blasts, VRAC inhibition or genetic depletion of *LRRC8A* led to impaired T-cell activation, cytokine production, and proliferation, specifically during weak TCR signaling conditions. Additionally, RNA sequence analysis of OT-I CD8^+^T cells activated by different TCR agonists revealed distinct transcriptional profiles in wild-type and KO cells at various TCR signal strengths, indicating a remarkable modulation of T cell activation and function by LRRC8A. Furthermore, LRRC8A deficiency in acute lymphocytic choriomeningitis virus infections, resulted in abnormal CD8^+^T cell-mediated antiviral immunity. Moreover, TCR sequencing of thymocytes revealed a highly diversified TCR repertoire in *LRRC8A*^f/f,Cd4-Cre^ mice, presumably due to weaker TCR signaling by self-peptides during thymic selection. Using a hypotonic environment to mimic the osmotic swelling elicited by osmolyte accumulation during T-cell activation, proximal signal defects in LRRC8A-deficient cells were found to occur owing to reduced molecular density and interactions resulting from cell volume enlargement. During T-cell blasts, *LRRC8A*-dependent RVD provides molecular density maintenance to counteract the decay of TCR signals. This review demonstrates that the activation and function of T cells depend on appropriate cell volume regulation. Moreover, we elucidated the mechanism of LRRC8A-associated cell volume regulation during T-cell activation and identified its physiological role in shaping the T-cell repertoire and enhancing antiviral immunity. Along with TCR and cGAS-STING signals, further exploration is required to determine whether LRRC8A mediates other classical immune signals that affect immune responses 30.

**Figure 2:**
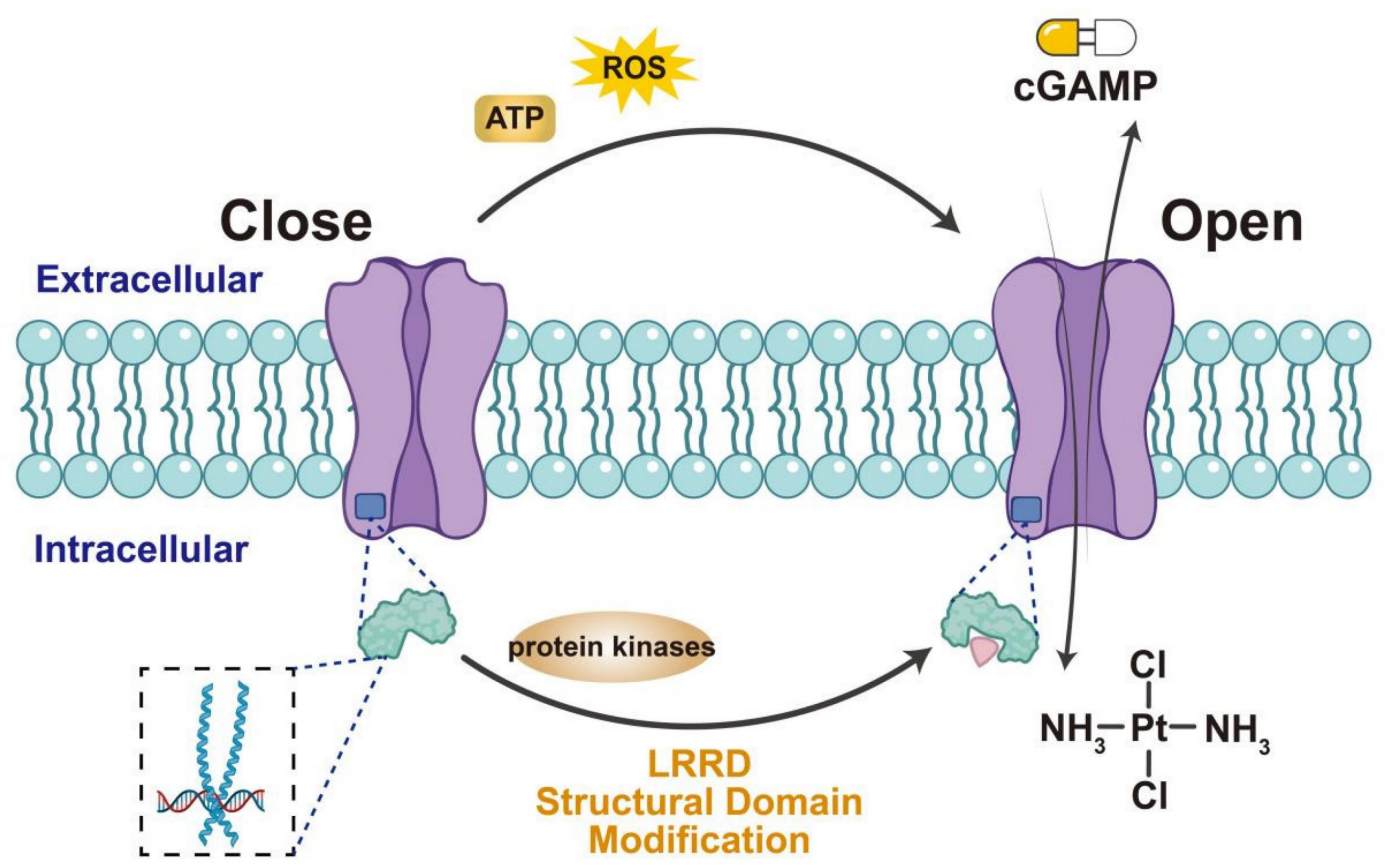
** Methods of activating LRRC8A/VRAC.** Extracellular ATP, ROS can stimulate VRAC to open the channel, as can the structural modification of LRRD at the end of LRRC8A and the use of various protease inhibitors may also stimulate VRAC activation. Activated VRAC promotes the release of cisplatin and cGAMP, making them important targets for the uptake of anticancer drugs.

VRAC activation facilitates the transport of immune-stimulating factors and T-cell activation, making it valuable for viral immunotherapy (Figure 2). Therefore, understanding the VRAC activation mechanism is crucial and can be further divided into two interconnected aspects: (1) identifying the protein domains crucial for channel activation and (2) determining the relevant cellular stimuli for VRAC activation. Unlike connexin, the distinct structural features of the LRRC8 subunit include an extended C-terminal cytoplasmic leucine-rich repeat domain (LRRD), a spatial shift at the C-terminus 41, and the exposure of residues during heteromeric chimerism, all of which contribute to VRAC inactivation 42,43. VRAC activity also depends on the release of reactive oxygen species 44-47 and adenosine triphosphate (ATP) 48-50 in numerous cellular systems and the phosphorylation of tyrosine 51-55. Therefore, domain modifiers and protein kinase inhibitors may serve as activators of VRAC alongside cGAMP adjuvants, TCR, or STING agonists to enhance antiviral immunotherapy.

## 5. Application of LRRC8A/VRAC in tumor adjuvant therapy

VRAC is a potential target for cancer therapy. Most reports have focused on alterations in LRRC8A in tumor cells, indicating its involvement in cancer cell proliferation, migration, death, and multidrug resistance through numerous signaling pathways 56. Additionally, ongoing research on the role of LRRC8A in immunity indicates that regulating VRAC activity and activating immune cells for antitumor effects may be a novel therapeutic strategy.

In colon adenocarcinoma cell lines, LRRC8A: C/E channel activity can selectively increase or decrease extracellular cyclic dinucleotide (CDN) signaling to STING, potentially enhancing the antitumor response 26. Another study observed that the potential of VRAC-associated transport of cisplatin was influenced by serum and inflammatory factors, such as TNF-α and IL-1β. This regulation primarily activates VRAC channels through cGAS, facilitating the transport of cGAMP into cells to activate STING. Serum and inflammatory factors can translocate cGAS to the cytoplasmic membrane and bind to phosphatidylinositol 4,5-bisphosphate (PIP2), anchoring it to the cell membrane to facilitate VRAC-associated transport of cisplatin 28. These studies used lung cancer and colon adenocarcinoma cell lines, suggesting that VRAC channel activation can facilitate immune responses, potentially enhancing the antitumor effects of cisplatin. Furthermore, Planells *et al.* found that the loss of the LRRC8A and LRRC8D subunits of VRAC increased resistance to clinically relevant cisplatin/carboplatin concentrations 57. Therefore, the subunit specificity and activity of VRAC may be involved in cisplatin transport, developing strategies to target VRAC may help overcome tumor resistance to cisplatin. Alongside chemotherapeutic drugs, such as cisplatin, which can activate VRAC to promote cell death, tumor DNA released by chemotherapeutic drugs can activate the cGAS-STING pathway through LRRC8A/VRAC to promote antitumor immunity. Reportedly, VRAC regulates the activation of the NLR family pyrin domain-containing protein 3 (NLRP3) inflammasome by regulating the efflux of itaconic acid, an oxidative stress indicator, and mitochondrial function, which is essential for antitumor immunotherapy 58. Therefore, VRAC is a potential target for drug screening and biomarker studies, offering promise for the development of new cancer treatment strategies. However, the effectiveness of the LRRC8A-mediated T-cell response in eliminating tumor cells remains unclear. If proven effective, this could be attributed to VRAC activation, which facilitates T-cell cytokine secretion or enhances T-cell recognition by tumor cells. However, further investigation and assessment are required to elucidate the specific mechanism underlying this phenomenon.

The aforementioned studies demonstrated that LRRC8A/VRAC activation can mediate T-cell responses and antigen presentation, enhancing antitumor effects in lung cancer 29. However, the relative conclusion lacks in vitro and in vivo experimental evidence.

**Figure 3:**
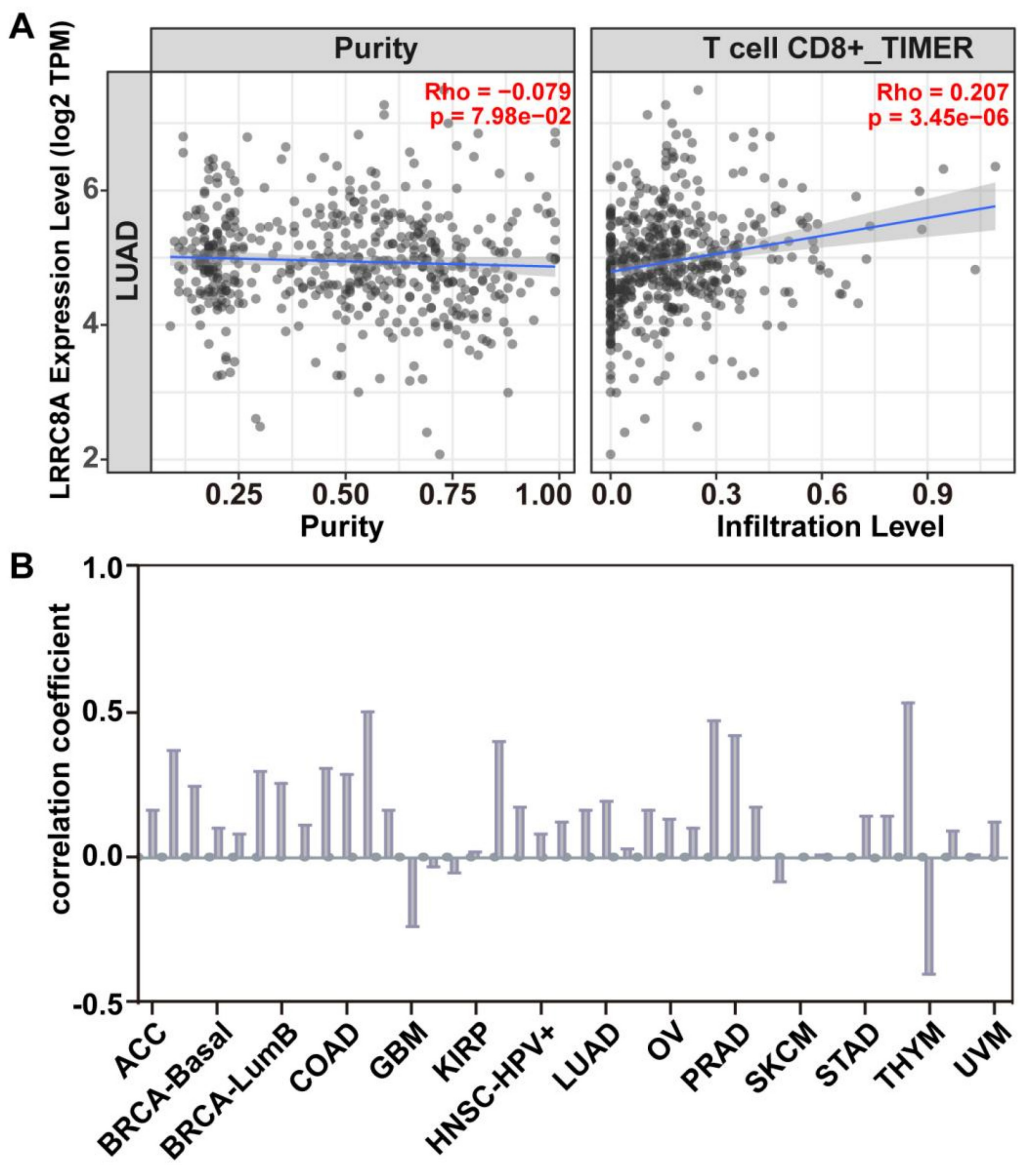
** Bioinformatics prediction of the correlation between LRRC8A expression and CD8^+^T cell infiltration.** The correlation between LRRC8A expression and the degree of CD8^+^T cell infiltration in various types of cancer was determined using the TIMER database. (A) In lung adenocarcinoma, the increased expression of LRRC8A correlated with increased CD8^+^T cell infiltration. (B) Among the 14 cancer types, 11 showed a positive correlation between LRRC8A expression and the degree of CD8^+^T cell infiltration. This suggests that LRRC8A may promote an immune response.

Based on current research, we analyzed the correlation between LRRC8A expression and CD8^+^T cell infiltration in lung cancer (Figure 3A) using the TIMER database 59, 60. Bioinformatic results indicated that LRRC8A facilitated CD8^+^T cell infiltration, consistent with previous findings in lung cancer research 28. Additionally, the correlation between LRRC8A and CD8^+^ T-cell infiltration in various tumors, including lung cancer, breast cancer, cholangiocarcinoma, and 14 other tumor types (Figure 3B), indicated that increased LRRC8A expression corresponded to higher levels of CD8^+^T cell infiltration in tumors. Based on our review and health information predictions, it is preliminary concluded that LRRC8A functions as an immune stimulator and holds promise as a novel immunotherapeutic target.

In summary, LRRC8A participates in cancer cell proliferation and migration, cellular immune responses and the entry of chemotherapeutic drugs such as cisplatin or cGAMP, into cancer cells to induce cell death. Additionally, LRRC8A promotes the infiltration of immune cells, which can regulate processes such as immune cell activation, inflammatory responses, and cytokine production 28,30. Thus, LRRC8A plays a crucial role in antitumor immunity.

### 6. Other immune cells

LRRC8A not only mediates innate responses of B and T cells. Vincent Compan *et al.* first associated LRRC8A with innate immunity 61. Pharmacological experiments have revealed that clinically approved and widely used nonsteroidal anti-inflammatory drugs suppress NLR family pyrin domain containing 3 (NLRP3) inflammasome activation by inhibiting the VRAC activity in macrophages 62. This study confirms that the regulatory effect of VRAC on the NLRP3 inflammasome may facilitate the establishment of a novel research field for understanding the pathogenesis of Alzheimer's disease, atherosclerosis, and other NLRP3-associated diseases. LRRC8A and VRAC may be involved in the activation of the NLRP3 inflammasome through various mechanisms, including the regulation of itaconic acid efflux and mitochondrial function 59,63. However, the importance of LRRC8A in different types of NLRP3 inflammasome activation signals, such as low osmotic pressure and DAMPs, may vary. Further research is required to fully understand the specific details and biological significance of these mechanisms.

## 7. Outlook

LRRC8A is crucial for T cell development and function, demonstrating favorable outcomes in antiviral and tumor immunity 10,26,28. However, research on LRRC8A and immunity remains in its preliminary stages, and understanding the intricate interactions between LRRC8A and the immune system is essential for developing highly precise and effective treatment approaches 63. Future research on LRRC8A as a novel immunologically relevant target should focus on several factors, as summarized below.

### 7.1 Exploring key signaling molecules regulating LRRC8A

The role of LRRC8A in immune cells is evident; however, the mechanisms underlying its ion-channel activity remain unclear 4,5,12. LRRC8A-mediated cell volume regulation influences the TCR signaling pathway by maintaining molecular density at a relatively constant level, thereby preventing excessive reduction in density and mitigating signal transduction during T-cell blasts 30. Additionally, LRRC8A affects immune function by transporting immune transmitters, such as cGAMP 26,29. To understand the biological processes associated with LRRC8A, a Kyoto Encyclopedia of Genes and Genomes pathway analysis was performed 30, revealing significant enrichment in pathways, including cytokine-cytokine receptor interaction, mitogen-activated protein kinase, rat sarcoma, T helper 17 (Th17) cell differentiation, Janus kinase/signal transducers and activators of transcription, STAT1/STAT6, and IL-2 signaling 30. Each of these signaling pathways should be further analyzed to reveal the mechanism by which LRRC8A affects T-cell function.

### 7.2. Assessing factors affecting LRRC8A function within the immune microenvironment

Aside from the signaling pathways that may affect T-cell function through LRRC8A, factors within the tumor microenvironment (TME) can hinder ion channel function 64. The adverse effects of the metabolic (hypoxia, adenosine, and electrolyte imbalance) and cellular [programmed cell death ligand 1 (PD-L1)] components of the TME can influence the antitumor potential of T cells through ion channels 65-67. Kv1.3 (K^+^ channel) overexpression corrected the immunosuppressive K^+^ accumulation in tumor necrotic areas, increased IL-2 and IFN-γ production, reduced tumor burden, and extended survival in mice with melanoma 68. Ion channels counteract TME's immunosuppressive effects on immune cells, restoring antitumor immunity 64. Thus, understanding LRRC8A activity mechanisms may contribute to developing effective treatments, specifically for patients' immune to conventional immunotherapies.

### 7.3. Exploring novel LRRC8A-based novel immunotherapy strategies

Modulating ion channel function could potentially elevate antitumor immune responses in immune cells, thereby enhancing immunotherapy 63. Novel immunotherapeutic treatments can be developed by combining ion channel regulation with traditional cancer therapies 28. Thus, through these integrated approaches, the therapeutic potential of ion channels in cancer immunotherapy can be maximized. Additionally, altering ion channels may enhance the susceptibility of cancer cells to the lethal effects of radiation or chemotherapy, thereby enhancing tumor control 28. The administration of chemotherapy and the transient receptor potential vanilloid 1 activator capsaicin resulted in a synergistic effect, enhancing apoptosis and inhibiting tumor cell migration 69. Immune checkpoint inhibitors, such as cytotoxic T-lymphocyte-associated proteins and programmed cell death proteins, combined with ion channel-targeted treatments can enhance cancer clinical outcomes 70. Ion channels, especially K^+^ channels, affect the production and function of immune checkpoint molecules. In animal experiments, this integrated approach demonstrated improved tumor control and prolonged lifespan 71, offering an enhanced solution for cancer treatment. Chronic alcohol consumption increases the population of peripheral blood lymphocyte regulatory T-cells (Tregs) and splenic Tregs in hepatitis B virus (HBV) mice by activating HBV X protein/specificity protein 1 (HBx)/(Sp1)/LRRC8A/ arachidonic acid signaling, indicating a correlation between LRRC8A and viral immunotherapy 72. Therefore, LRRC8A is a potential immune target against tumor growth and viral infections. Novel LRRC8A-based immunotherapeutic strategies hold promise as innovative approaches for the potential treatment of cancer and viral infections.

### 7.4. Challenges in targeted drug development

The effectiveness of ion channel-targeted therapeutics depends on the development of potent and selective ion channel modulators 63. Thus, it is crucial to develop drugs that specifically target ion channels in immune cells while minimizing adverse effects on healthy cells. LRRC8A enhances T-cell function and is anticipated to be a crucial target for activating immunity. Currently, there are numerous reports on VRAC inhibitors such as 4-(2-Butyl-6,7-dichloro-2-cyclopentyl-indan-1-on-5-yl) oxobutyric acid (DCPIB) and dicumarol 73,74. However, data regarding VRAC agonists, specifically LRRC8A, is limited. Sphingosine-1-phosphate, guanosine triphosphatase, ATP, and angiotensin can activate the VRAC channels; however, they fail to activate LRRC8A specifically 74,75. m5C modification enhances LRRC8A mRNA stability and upregulates its expression, followed by activation of the PI3K/AKT signaling pathway 76. Increased oxidative stress can either directly induce post-translational modifications of ion channels or indirectly modulate channel activity by affecting the signaling pathways that regulate gene transcription, protein trafficking, and turnover 77. These studies suggest that LRRC8A expression enhancement in immune cells could facilitate T-cell function. Additionally, the development of antibodies and small-molecule drugs targeting ion channels, such as those of K^+^ and Ca^2+^, has shown promise 78,79. Their advantages include high specificity, low off-target effects, and tunable in vivo half-lives 80. Despite significant progress, challenges persist in the development of LRRC8A agonists that require a multidisciplinary approach, involving chemistry, bioinformatics, bioengineering, and biophysics.

## 8. Conclusion

VRAC, a member of the ion channel family, regulates cell volume by mediating Cl^-^ efflux. It is widely expressed in mammalian cells and comprises five family members (LRRC8A-E). LRRC8A is the only obligatory subunit of the VRAC. Additionally, LRRC8A-formed VRAC, which functions through the Cl^-^ channel, has been reported to transport small molecules such as cGAMP, cisplatin, GABA, and taurine. LRRC8A is involved in T lymphocyte activation and innate immunity. Understanding the intricate interactions between LRRC8A and the immune system may lead to the development of novel treatment approaches that effectively address tumor growth and viral infections; however, the ion channel realm is complex. Although significant progress has been made in understanding the correlation between LRRC8A and the immune system, limited data exists regarding these mechanisms. Additionally, exploring novel immunotherapeutic strategies using LRRC8A-targeted medicines is necessary because certain traditional treatments, such as chemoradiotherapy drugs, immune checkpoint inhibitors, and ion channel-targeted medicines, may exhibit synergistic effects and enhance therapeutic results. It is crucial to elucidate the limitations and challenges associated with LRRC8A-targeted immunotherapies. A thorough assessment is necessary for off-target and possible adverse effects. LRRC8A is a promising and developing field requiring further investigation. We anticipate that future research will explore the potential role of LRRC8A in immune function and offer significant solutions for disease treatment 63.
